# Analysis of cell surface markers specific for transplantable rod photoreceptors

**Published:** 2013-09-28

**Authors:** Kai Postel, Jessica Bellmann, Victoria Splith, Marius Ader

**Affiliations:** DFG-Center for Regenerative Therapies Dresden (CRTD), Cluster of Excellence, Technische Universität Dresden, Dresden, Germany

## Abstract

**Purpose:**

Transplantation of cells into retinas affected by degenerative diseases to replace dying photoreceptors represents a promising therapeutic approach. Young photoreceptors of 4-day-old mice show the highest capacity to integrate into the retinas of adult mice following grafting. Additional enrichment of these donor cells before transplantation with cell surface marker–dependent sorting methods further increases success rates. Currently, defined cell surface markers specific for transplantable photoreceptors that can be used for enrichment are limited. Therefore, identifying alternative targets would be advantageous.

**Methods:**

Microarray data of young rod photoreceptors were analyzed using the Database for Annotation, Visualization and Integrated Discovery combined with a literature search to identify genes encoding for proteins containing extracellular domains. Candidate genes were further analyzed with reverse transcriptase polymerase chain reaction (RT–PCR) for their retinal specificity. In situ hybridization and immunohistochemistry were used to identify their localization within the retina.

**Results:**

Enrichment of candidates by Database for Annotation, Visualization and Integrated Discovery revealed 65 proteins containing extracellular domains. Reverse transcriptase polymerase chain reaction identified Atp8a2, Cacna2d4, Cadm2, Cnga1, Kcnv2, and Pcdh21 as expressed in the retina and only a few additional tissues. In situ hybridization and immunohistochemistry showed specificity of Cacna2d4, Kcnv2, and Pcdh21 for photoreceptors in the retinas of young mice.

**Conclusions:**

Cacna2d4, Kcnv2, and Cnga1 were identified as specific for target cells in the retinas of young mice and could serve as candidates for rod photoreceptor enrichment to replace cells in retinal degenerative diseases.

## Introduction

Retinitis pigmentosa and age-related macular degeneration are groups of degenerative diseases of the retina leading to the death of photoreceptors. Most forms of retinitis pigmentosa are hereditary. More than 60 disease-causing genes have been identified (GHR). Age-related macular degeneration is a multifactorial disease with age, smoking, and genetic predisposition identified as major risk factors. Development of therapies addressing the broad range of factors causing such diseases is highly challenging. A general approach might be a cell replacement strategy in which degenerated photoreceptors are replaced by transplantation of healthy cells.

Recent preclinical studies provided evidence that transplantation of rod photoreceptors, isolated from the developing mouse retina, into the sub-retinal space of wild-type mice results in integration of donor cells into the host outer nuclear layer (ONL) and the formation of morphological mature photoreceptors [1-3]. Interestingly, the first studies suggested restoration of some visual function upon transplantation into mouse models of retinal degeneration [4-6] with rod photoreceptors isolated around postnatal day (P) 4 showing the highest integration efficiency [1,3].

The purification of transplantable donor photoreceptors by cell surface markers would be advantageous in future clinical applications avoiding the need for genetic manipulations of donor cells for the expression of cell type–specific reporter genes. Indeed, enrichment of rod photoreceptors by magnetic associated cell sorting (MACS) significantly increased the number of integrated cells [7]. For this technique, the glycosylphosphatidylinositol (GPI)-anchored cell surface molecule ecto-5′-nucleotidase (Nt5e, also termed CD73), expressed by photoreceptors in retinas of 4-day-old mice [7,8], was used. Nt5e is bound by a primary anti-CD73 antibody, which is detected by a secondary antibody coupled to a magnetic bead. This allows the magnetic separation of photoreceptors from other retinal cells and the subsequent transplantation of purified photoreceptors. For a clinical application in humans, cell sources other than primary retinal cells have to be considered. Human and murine embryonic stem [9,10] as well as induced pluripotent stem cells [11,12] were recently demonstrated to differentiate in vitro into photoreceptor-containing cell populations. Here, purification of photoreceptors before transplantation might be important to increase integration rates and to prevent tumor formation caused by contaminating undifferentiated cells [13]. In the retinas of 4-day-old mice, CD73 is specific for rod photoreceptors [14], but expression in other tissues, including renal progenitor cells [15], natural killer cells [16], or mouse embryonic stem cells [17], was recently reported. Therefore, screening for further cell surface markers specific for transplantable photoreceptors might be advantageous for effective purification of donor cells derived from pluripotent stem cell cultures.

Here, we analyzed in situ the expression of genes encoding cell surface proteins that are characteristic of rod photoreceptors at P4, the optimal ontogenetic stage for successful transplantation. Microarray experiments identified genes that are higher expressed in P4 rod photoreceptors (GSE29318; see also [7]). The received entity list was analyzed using Database for Annotation, Visualization and Integrated Discovery (DAVID). Additionally, a literature search of interesting candidates was performed, and promising genes were tested for their tissue and cell type specificity in 4-day-old and adult mouse retinas with reverse transcriptase polymerase chain reaction (RT–PCR), in situ hybridization (ISH), or immunohistochemistry.

## Methods

### Mice

C57BL/6j mice were purchased from Janvier (Le Genest-Saint-Isle, France) and used for RT–PCR and ISH. Mice expressing enhanced green fluorescent protein (EGFP) under the control of the neural retina leucine zipper (Nrl) promoter (Nrl-EGFP mice [18]) were used for immunohistochemistry (kind gift of Anand Swaroop; National Eye Institute, Bethesda, MD). All animals were treated in strict accordance with European Union and German laws (Tierschutzgesetz) and the Association for Research in Vision and Ophthalmology Statement for the Use of Animals in Ophthalmic and Vision Research.

### Microarray and Database for Annotation, Visualization and Integrated discovery analysis

Microarray data were published in [7] and deposited in the National Center for Biotechnology Information’s (NCBI; Bethesda, MD) Gene Expression Omnibus (GEO) [19] where they are accessible through GEO Series accession number GSE29318. Data were further analyzed with DAVID [20], and entities were enriched with the gene ontology term “topological domain: extracellular” to receive potential extracellular targets.

### Complementary deoxyribonucleic acid synthesis and reverse transcriptase polymerase chain reaction

Total RNA was isolated from the brain, heart, liver, kidney, spleen, lung, muscle, thymus, and retina of 4-day-old mice with the Qiagen RNeasy Mini Kit and QIAshredder following the manufacturer’s instructions (Qiagen, Hilden, Germany). Subsequent cDNA synthesis was performed with SuperScript II reverse transcriptase (Life Technologies, Darmstadt, Germany). Qiagen HotStarTaq Plus Mastermix Kit was used for tissue-specific gene expression studies. Specific primers were designed using the Basic Local Alignment Search Tool (BLAST) from NCBI. Accession numbers and primer sequences used in each PCR experiment are summarized in Table 1. Succinate dehydrogenase complex, subunit A (SDHA) was used as the housekeeping gene.

**Table 1:** Primer list

**Accession Number**	**Gene**	**Forward primer (5′ to 3′)**	**Reverse primer (5′ to 3′)**	**Amplicon length (bp)**
NM_026142.4	*3632451O06Rik*	AACAAAGCCACCACATGGGA	CCAGCTTCTCACCAGACCTT	1069
NM_178745.4	*6330442E10Rik*	GACTGTGATAAGAGGTCCCAGT	GAAACACCACCGAACACACG	1218
NM_007405.2	*Adcy6*	TTGGCGCCAGCCAAAAACGG	AGCCAAGCCATGGACGCCAA	968
NM_015804.3	*Atp11a*	TGACAGCTTGCACGCCACGA	ATCCAAGCCTCTGCACGCCT	953
NM_015803.2	*Atp8a2*	TCAACGAAGAGCTGGGGCAGGT	ATGGCCGCCCTTGTTGCATCT	985
NM_007568.5	*Btc*	TGCACAGGTACCACCCCTAGACA	GCACATCAGCTTTGACTCTGGGTCC	1100
NM_001033382.2	*Cacna2d4*	TGCCACTGGACAAAGGGAAGCG	TTCGGCCGCACTGTGGTGAT	995
NM_178721.4	*Cadm2 Variant 1*	AAAGTCAAAGGCAGCCAAGGGCA	AGCATGGTCAGGGCCATTCTGG	933
NM_019707.4	*Cdh13*	TGCGTCCTGCTGTCCCAGGT	CAGCAGCGCAGGTGACACCA	924
NM_007723.2	*Cnga1*	AACATCCTCCCCTGCCAATGCCAT	GCAGATCGGTCGGTATCACTGACA	994
NM_172338.2	*Dnajc16*	TTGGGCGTGTCCTGGCGATT	TTGGGCGTGTCCTGGCGATT	1011
NM_013565.2	*Itga3*	TGCGCATGAGGCATTGCTCACCCT	ACGGCCACAAGCACCAACCACA	993
NM_183179.1	*Kcnv2*	TTTGCACGCAGCGCCCTCAA	AGCTCCTGGTGTGACGCTCCAT	1061
NM_172668.3	*Lrp4*	AACGCAACTGCACCACCTCCA	TCAGCAATGATGCGACGCCCA	997
NM_130878.2	*Pcdh21*	TCCGCGGAGACATGAGGCGT	GGCATTGGCTCCCTGGTCTGAG	1193
NM_023281.1	*Sdha*	ATGCTGTGGTTGTAGGCGCT	TTCCCCAGAGCAGCATTGAT	151
NM_025491.3	*Susd3*	AATGGGAGCACCGTTGACTGGT	TCCAGCTGGGCAAACGTTGACT	623

Primers that were used to evaluate expression of candidate genes in different PN4 mouse tissues are listed with their accession number, gene symbol, sequence and amplicon length.

### Eye preparation and cryosectioning

Eyes of 4-day-old and adult mice were fixed in 4% paraformaldehyde (PFA; VWR, Darmstadt, Germany) overnight and dehydrated with 30% sucrose (VWR) solution. Lens and vitreous body were removed, and the eyes were embedded in tissue freezing medium (VWR) and frozen in a tank filled with 2-methyl-butane (VWR) placed in liquid nitrogen. The retinas were sectioned at 20 µm thickness with a Leica CM1900 cryotome (Leica Biosystems, Nussloch, Germany).

### In situ hybridization

Digoxigenin-labeled RNA probes were prepared following instructions of the TA Cloning Kit Dual Promoter (Life Technologies). Genes of interest were cloned into pCRII vector (Life Technologies) and transformed into One Shot TOP10 *E. coli*. Amplification of sense and antisense RNA probes was performed with T7 and SP6 RNA polymerase (Roche, Grenzach-Wyhlen, Germany), and the probes were subsequently purified with the RNeasy Mini Kit (Qiagen). A stock solution of 20X saline sodium citrate buffer (SSC; 175.3 g NaCl [Sigma-Aldrich, Munich, Germany], 88.2 g sodium citrate, 0.1% diethylpyrocarbonate [DEPC], [both AppliChem, Darmstadt, Germany]; in 1 l deionized water) was prepared. Cryosections were incubated with specific antisense and sense RNA probes diluted 1:2,000 in prewarmed hybridization buffer (25% 20X SSC, 1 ml/l Tween 20 pure [SERVA, Heidelberg, Germany], 50% formamide [Sigma-Aldrich], 1 mg/ml RNA from torula yeast, Type VI, 0,1 mg/ml Heparin, 2% Denhardts solution [50X; all Sigma-Aldrich], 1 ml/l 3-[(3-cholamidopropyl)dimethylammonio]-1-propanesulfonate (CHAPS), 0,1 g/ml Dextran [all Carl Roth, Karlsruhe, Germany], 10 mM EDTA [VWR]) overnight at 70 °C. The 20X SSC was diluted for washing steps into 5X SSC (3 times washing for 1 h, 1 time washing overnight; 500 ml formamide, 250 ml 20X SSC; 10 ml 10% Tween; 240 ml DEPC-H_2_O) and 2x SSC (2 times washing for 1 h; 200 ml formamide, 40 ml 20X SSC + 4 ml 10% Tween, 156 ml DEPC-H_2_O) SSC. Additional washing with maleic acid buffer (MAB; 100 mM maleic acid pH 7.5 with NaOH [both Sigma-Aldrich], 150 mM NaCl, 0.1% Tween) and blocking for 1 h at room temperature in MAB containing 1% blocking reagent (Roche) was performed before incubation with antidigoxygenin antibody (1:5,000 in blocking solution, overnight, 4 °C). Slides were washed 5 times for 10 min in MAB and 2X in AP buffer (100 mM Tris [Carl Roth], pH 9.5, 50 mM MgCl_2_ [VWR], 100 mM NaCl, 0.1% Tween) at room temperature. For signal development, slides were incubated in BM Purple (Roche) until a purple signal became visible. Slides were subsequently washed with PBS (Life Technologies, pH 7.4, did not contain magnesium, calcium or phenol red) containing 5 mM EDTA and mounted on glass slides.

### Immunohistochemistry

For antibody staining, eyes were fixed for 4 h in 4% PFA. After cryosectioning, the slides were rehydrated in PBS for 30 min, blocked for 30 min in blocking solution containing PBS, 5% goat serum (Sigma-Aldrich), 1% bovine serum albumin (BSA; Sigma-Aldrich), and 0.3% Triton X-100 (TU-Dresden Pharmacy, Dresden, Germany), and incubated overnight with rabbit anti-Pcdh21 antibody (kind gift of Amir Rattner; Jeremy Nathans laboratory at the Johns Hopkins University, Baltimore, MD) in a 1:50 dilution in PBS and 1% BSA. Visualization of primary antibody was achieved by 2 h incubation with secondary Cy3-conjugated goat anti-rabbit antibody (Jackson ImmunoResearch, Suffolk, UK). Samples were counterstained with 4',6-diamidino-2-phenylindole dihydrochloride and mounted on glass slides.

### Graphic images

Gel images were taken with the QUANTUM gel documentation system model 1100 (PEQLAB Biotechnology, Erlangen, Germany) and processed with Adobe Illustrator (Adobe Systems Software, Dublin, Ireland). ISH and fluorescence images were taken with a fluorescence microscope (Z1-Imager with ApoTome; Carl Zeiss Meditec, Jena, Germany), processed with ImageJ (National Institutes of Health) and Adobe Photoshop, and arranged with Adobe Illustrator (Adobe Systems Software).

## Results

### Identification of cell surface markers specific for young rod photoreceptors

To identify genes specific for young rod photoreceptors and encoding proteins located at the surface of the target cells, we analyzed microarray data sets generated recently in our group [7]. Seven hundred forty-four genes were identified to be more than twofold higher expressed in rod photoreceptors at P4 compared to the other retinal cell types. These genes were analyzed with DAVID. Enrichment with the gene ontology term “topological domain: extracellular” resulted in a list containing 63 genes (Supplemental Table 1). Additionally, cadherin 13 (Cdh13) and potassium voltage-gated channel subfamily V member 2 (Kcnv2) were added to the candidate list as they showed more than 2.5-fold upregulation and are known to have extracellular domains [21,22] but did not show up during DAVID analysis. A literature search was performed with all entities using publicly available platforms, including pubmed, eurexpress, uniprot, biogps and others, to identify genes that are known (1) to be not ubiquitously expressed, or (2) to be expressed in the photoreceptor layer only, or (3) to have unknown expression patterns to identify new potential photoreceptor-specific genes.

### Expression of selected genes in different tissues of 4-day-old mice

**Figure 1:**
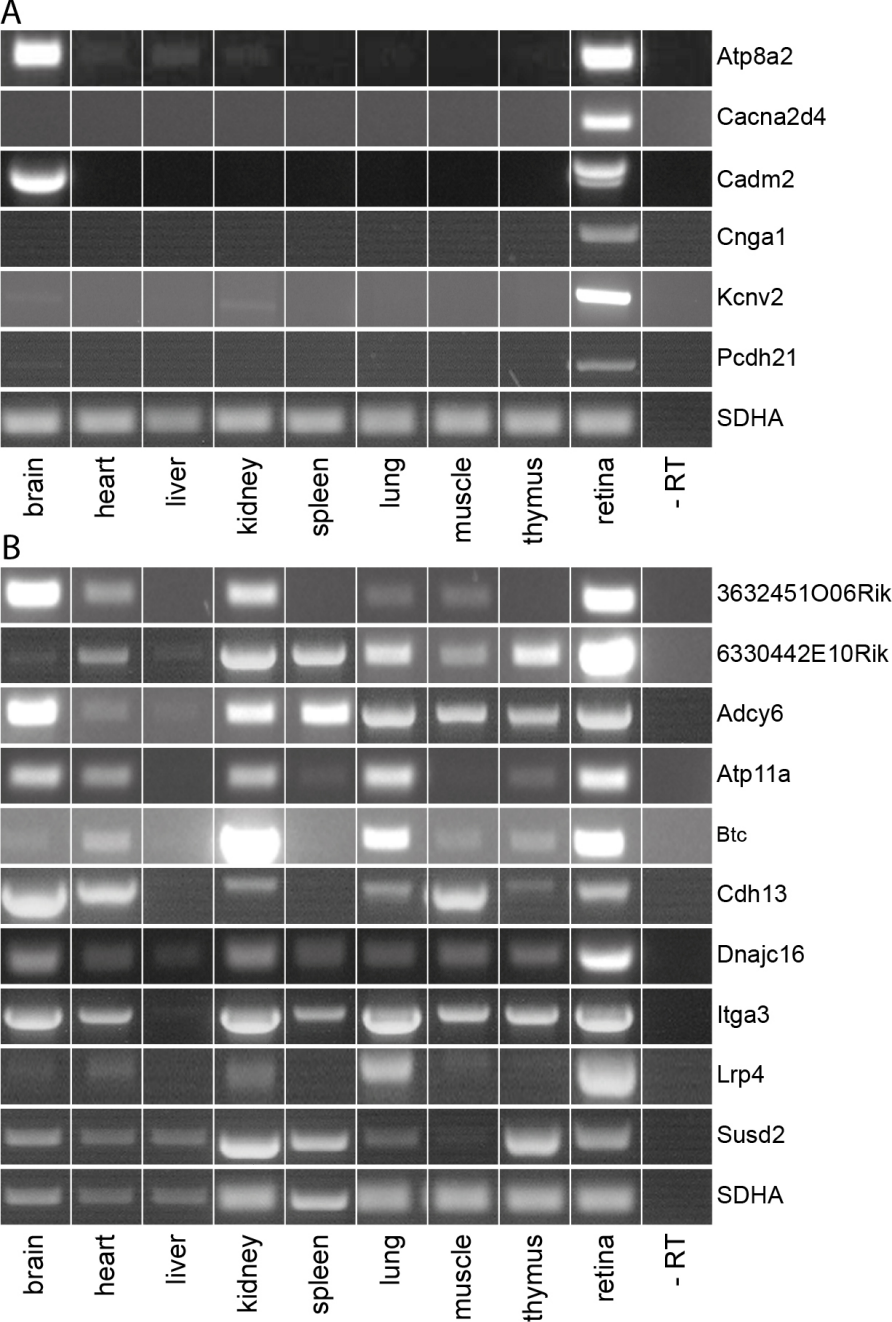
The expression of genes selected from microarray data sets and enriched with Database for Annotation, Visualization and Integrated Discovery was analyzed in different tissues of 4-day-old mice. Reverse transcriptase polymerase chain reaction shows that Atp8a2, Cacna2d4, Cadm2, Cnga1, Kcnv2 and Pcdh21 are expressed prominently in the retina and only a few additional tissues (**A**) whereas 3632451O06Rik/uncharacterized protein C14orf37 homolog, 6330442E10Rik/Tmem229b, Adcy6, Atp11a, Btc, Cdh13, Dnajc16, Itga3, Lrp4 and Susd2 are ubiquitously expressed (**B**). SDHA was used as a housekeeping gene.

Genes that were enriched with DAVID and selected from the literature search were further analyzed with RT–PCR for their distribution within different organs of 4-day-old mice (Figure 1). Cnga1 and Cacna2d4 were expressed in the retina only and thus might be retina specific whereas Pcdh21, Cadm2, and Atp8a2 showed additional expression in the brain. Kcnv2 showed an additional band in the kidney (Figure 1A). Although the primers were selected for high specificity, a second band showed up in the RT–PCR for Cadm2 that might represent the shorter transcriptional isoform two of the gene. All candidates shown in Figure 1A were selected for further investigation via ISH or immunohistochemistry. 3632451O06Rik/uncharacterized protein C14orf37 homolog, 6330442E10Rik/Tmem229b, Adcy6, Atp11a, Btc, Cdh13, Dnajc16, Itga3, Lrp4, and Susd2 were also identified from the microarray data sets as higher expressed in young photoreceptors than in the other evaluated retinal cell populations and might thus be specific for photoreceptors in the retina. RT–PCR for different tissues in the 4-day-old mice confirmed the high expression of these genes within the retina, but also revealed a wide distribution within the murine body (Figure 1B). Thus, they were excluded from further investigation.

### In situ hybridization in the retina of four-day-old mice and adult mice

**Figure 2:**
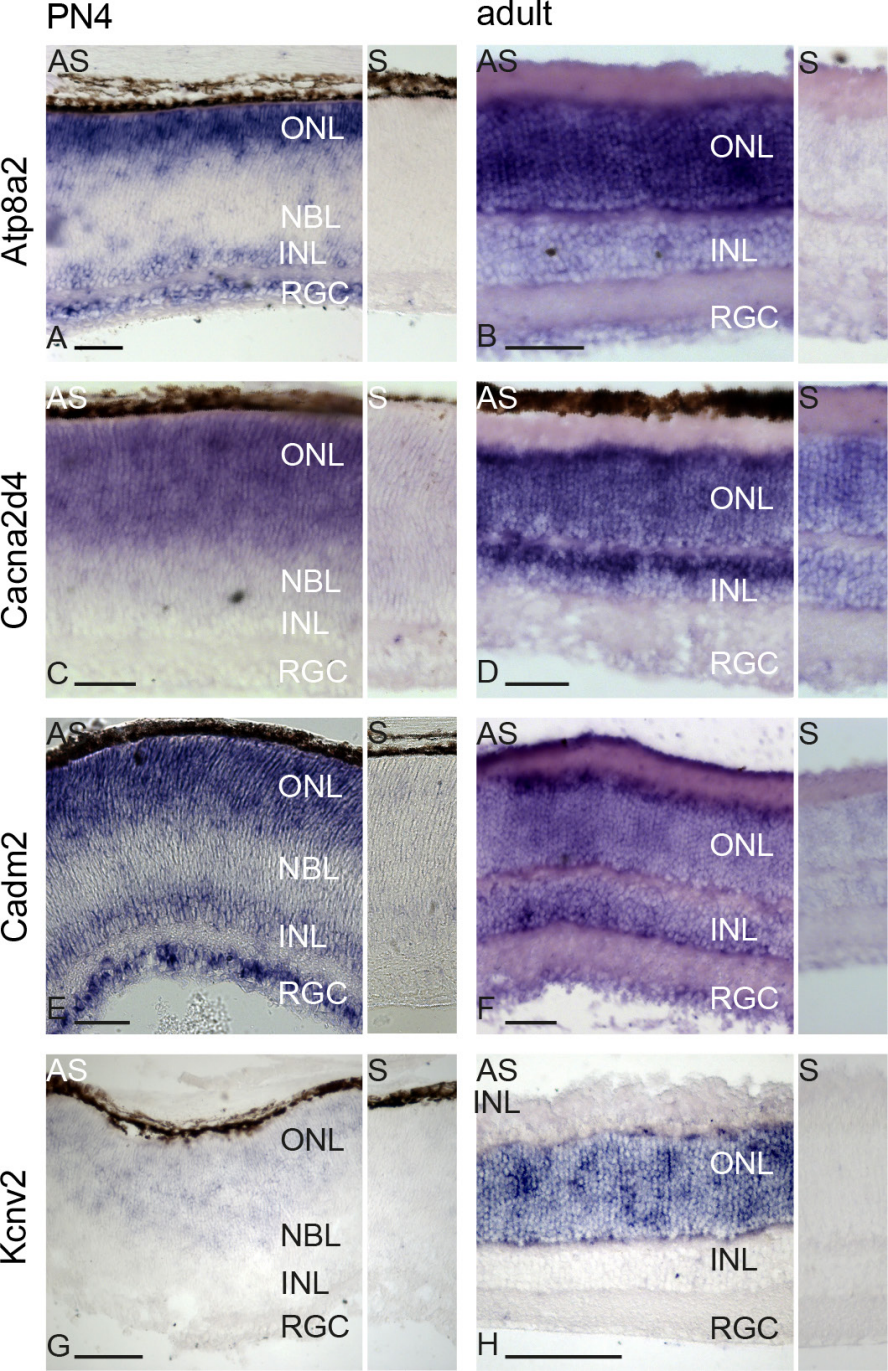
In situ hybridization displays the expression pattern of selected candidates in 4-day-old and adult mice. Cacna2d4 and Kcnv2 are expressed in the developing photoreceptor layer in 4-day-old retinas and in the ONL of adult retinas (**C**, **D**, and **G**, **H**). Cacna2d4 shows additional expression in the mature INL (**D**). Atp8a2 and Cadm2 are expressed in 4-day-old ONL, INL, and RGC (**A**, **E**). Expression throughout the retina is observed in adult animals (**B**, **F**). Attached smaller images represent the corresponding sense controls for each staining. Scale bar: 50 µm. AS: anti sense; INL: inner nuclear layer; NBL: neuroblast layer; ONL: outer nuclear layer; RGC: retinal ganglion cell layer; S: sense.

ISH was performed with selected genes that turned out to be highly expressed in the 4-day-old murine retinas and only a few additional organs (Atp8a2, Cacna2d4, Cadm2, Kcnv2; Figure 1A). Atp8a2 (Figure 2A) and Cadm2 (Figure 2E) were expressed in the developing inner nuclear layer (INL), ONL, and the retinal ganglion cell layer (RGC) in the retinas of 4-day-old mice. In adult mice, Atp8a2 (Figure 2B) and Cadm2 (Figure 2F) were expressed in the mature INL, ONL, and RGC, although Atp8a2 expression in the INL was weaker than in the ONL. Cacna2d4 (Figure 2C) and Kcvn2 (Figure 2G) were restricted to the developing photoreceptor layer in the retinas of the 4-day-old mice. In the adult mice, Kcnv2 (Figure 2H) expression was restricted to the ONL, while Cacna2d4 (Figure 2D) expression was also observed in the INL.

### Protocadherin-21 staining

**Figure 3:**
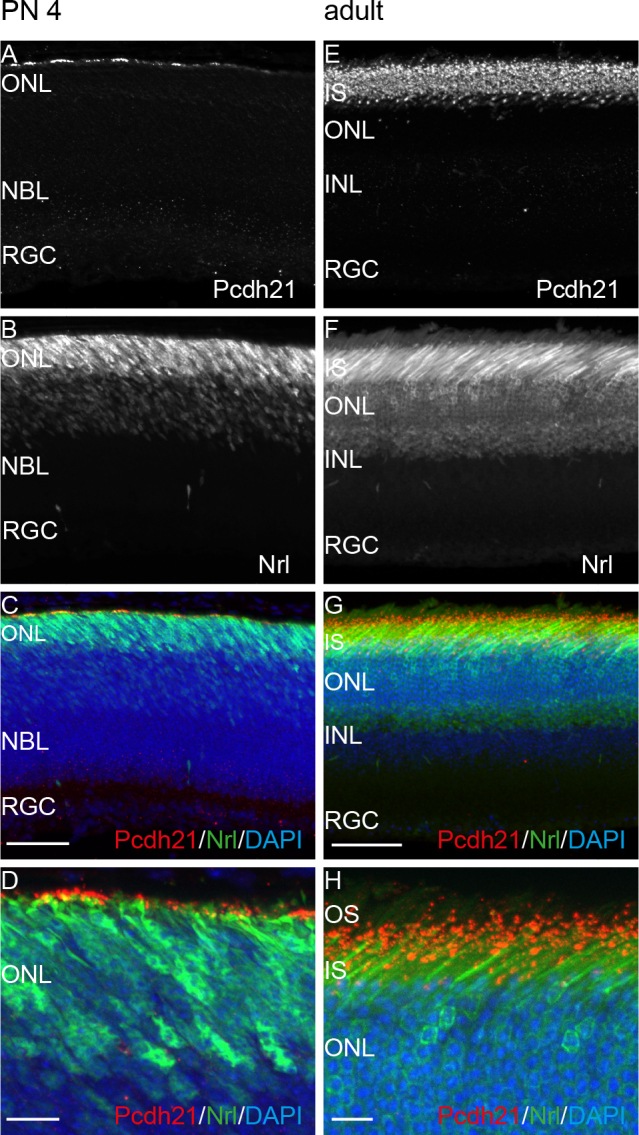
Pcdh21 staining occurs in photoreceptors in the retina of postnatal day 4 and adult mice expressing green fluorescent protein under the control of the neural retina leucine zipper promoter. Green fluorescent protein is expressed in developing (**B**) and mature (**F**) rod photoreceptors. Pcdh21 is localized at the tip of the developing inner segment in the retina (**A**, **C**, **D**) of 4-day-old mice. In the adult retina, Pcdh21 staining was observed at the connecting cilium between the inner and outer segments (**E**, **G**, **H**). **D** and **H** show magnifications of **C** and **G**. Scale bar in **C** representative for **A** and **B** and scale bar in **G** representative for **E** and **F**: 50 μm. Scale bars in **D** and **H**: 10 μm. INL: inner nuclear layer; IS: inner segments; NBL: neuroblast layer; ONL: outer nuclear layer; OS: outer segments; RGC: retinal ganglion cell layer.

Pcdh21 showed more than 3.5-fold upregulation in the microarray data sets, and RT–PCR analysis suggested high specificity for the retina in 4-day-old mice. Due to the availability of an extracellular binding antibody that could potentially also be used for MACS-mediated enrichment, Pcdh21 localization in mouse retinas was directly tested with immunohistochemistry (Figure 3). Nrl-EGFP mice [18] showing EGFP signals in developing (Figure 3B) and mature (Figure 3F) rod photoreceptors were used to visualize the photoreceptor layer. In the retinas of 4-day-old mice, Pcdh21 staining was restricted to the tips of developing photoreceptors (Figure 3A–D). A punctuated staining was observed at the connecting cilium region between the outer and inner segments of the photoreceptors in the retinas of adult mice (Figure 3E–H).

## Discussion

We analyzed 744 genes identified with microarray experiments and did subsequent enrichment with DAVID to specify the genes expressed in young, transplantable photoreceptors and encode for proteins localized to the cell surface. We excluded several genes with the literature search because they were ubiquitously expressed and are therefore not specific for young photoreceptors.

Atp8a2, Cacna2d4, Cadm2, Cnga1, Kcnv2, and Pcdh21 were identified with RT–PCR as expressed in the retina and only a few additional tissues. ISH and immunohistochemistry showed high specificity of Cacna2d4, Kcnv2, and Pcdh21 for photoreceptors in the retinas of 4-day-old mice.

Cacna2d4 is the subunit of a voltage-dependent calcium channel that regulates its activation and inactivation and the calcium current density [23]. Mutations in Cacna2d4 cause cone-rod dysfunction, and the corresponding mouse model showed a decrease in rod photoreceptors. However, the number of cone cells was not affected until 6 weeks of age. Additionally, substantial loss in the activity of retinal second-order neurons was observed [23] corresponding to the observation of our ISH analysis that showed expression of Cacna2d4 in the ONL and INL of adult mice. Although Cacna2d4 is expressed in the INL in adulthood, it represents a promising candidate for MACS-mediated photoreceptor enrichment due to its high specificity to photoreceptors in the 4-day-old murine retina and the large extracellular N-terminus suitable for generating extracellular binding antibodies.

Kcnv2 is a subunit of a voltage-gated potassium-channel and forms heteromultimers with other subunits. In response to depolarization, the channel selectively transfers potassium ions through the plasma membrane. RT–PCR from adult mice showed high expression in the heart and eye and lower expression in the lung, cerebellum, and cerebrum. ISH showed expression in the ONL of the adult mouse and in the eye of perinatal rats [24]. Kcnv2 was identified during a screening as the disease-causing gene for the retinal condition cone dystrophy with supernormal rod electroretinogram, and ISH was performed on human retinal tissue showing high levels of Kcnv2 expression in the photoreceptor layer [22]. Patients with cone dystrophy with supernormal rod electroretinogram showed reduced and delayed electroretinograms but an exaggerated rod response due to higher light intensities [22]. The alterations in cone signaling indicate that Kcnv2 is also expressed in cone photoreceptors. We also showed the restriction of Kcnv2 expression to the retina, brain, and kidney of 4-day-old mice and the high specificity of Kcnv2 to the developing photoreceptor layer of the 4-day-old retina. Although generating an extracellular binding antibody against Kcnv2 might be challenging because of its four short extracellular domains [22], it seems to be an interesting target for photoreceptor enrichment via MACS technology.

Pcdh21 was expressed in photoreceptors and localized to the base of the outer segments of cone and rod photoreceptors and at the edges of evaginating discs. Pcdh21 might play a role in protein trafficking between the inner and outer segments [25]. Using RT–PCR, we showed that Pcdh21 is expressed in the retinas and brains of 4-day-old mice and verified its localization to the apical tip of the developing segment in the 4-day-old retina. In addition, MAC sorting was tested with the Pcdh21 antibody but unfortunately did not lead to successful enrichment of young photoreceptors (data not shown). One reason for this finding might be the restricted localization of Pcdh21 to the tip of young photoreceptors and indicates that for successful MACS the target antigen needs to cover a bigger area of the cell surface.

In our RT–PCR analysis, Cnga1 expression was detected in the retinas of 4-day-old mice. Three units of Cnga1 and one unit of Cngb1 form together a cyclic guanosine monophosphate–gated cation channel in the plasma membrane of rod photoreceptors. This channel allows the hyperpolarization of the cells as the final step in the phototransduction cascade [26]. Cnga1 consists of cytoplasmic N- and C-termini, six membrane-spanning subunits, and a pore-forming loop. Due to high specificity to rod photoreceptors, Cnga1 might be a good target for MACS-mediated enrichment of young photoreceptors.

Several candidates identified with microarray analysis were tested in this study for their specificity to young rod photoreceptors in the retinas of 4-day-old mice. Cacna2d4, Kcnv2, and Cnga1 were identified as specific for young photoreceptors and therefore represent suitable cell surface targets for MACS-mediated enrichment of transplantable donor cells. Next steps should now involve designing and producing highly specific antibodies for these targets enabling enrichment and purification of photoreceptors. These antibodies should first be tested with primary retinal cells from transgenic mice containing rod photoreceptors expressing fluorescent reporter molecules to verify successful enrichment. Then this approach should be translated to photoreceptors generated from stem cell sources. Differentiation of embryonic stem or induced pluripotent stem cells into retinal cell types has been established by several groups [9-12], including the generation of transplantable rod photoreceptors that showed the potential to integrate into murine hosts [17]. In this study, the generated photoreceptors were enriched via fluorescence activated cell sorting mediated by viral infection with a construct containing rhodopsin promoter driving GFP expression [17]. Using a sorting approach independent from genetically encoded fluorochromes with Cacna2d4 or Cnga1, for example, to enrich in vitro generated photoreceptors would avoid the need for viral infection and genetic manipulation of donor cells, and lead to easier clinical application. Given the improved protocols for generating purely neural lineages including retinal tissue and photoreceptors [9-13,17] from pluripotent stem cells, antibodies against cell surface markers that are expressed in photoreceptors but additionally in several other tissues but not the brain (e.g., 6330442E10Rik, Btc, Lrp4) might also represent promising candidates for rod photoreceptor enrichment. Furthermore, a method for increasing the purity of enriched photoreceptor precursors and avoiding contamination by unwanted cell types might be a pull-down approach using two antibodies for sorting. Protocols have been established showing the feasibility of subsequent positive selections by MACS using two different antibodies (Miltenyi Biotec, Bergisch Gladbach, Germany [27]). The first sorting can be performed with an antibody directed against target cells such as rods and some other tissues (e.g., with anti-Kcnv2 antibody binding rods, cones, the brain, and barely the kidney) leading to enrichment of the corresponding cell populations. After the antibody is removed, a second sorting round could be applied using an antibody also directed against target cells and other opposing tissues (e.g., anti-Lrp4 antibody binding rod photoreceptors and other tissues but not the brain and only barely the kidney) allowing further depletion of unwanted cell types. This approach should result in an enriched photoreceptor population but would not require such a stringent selection of antibodies that solely bind to photoreceptors. Notably, the observation that the generation of neural cells in culture is an efficient process and that the differentiation into cell types of other tissues is low or absent makes a pull-down approach with two antibodies a suitable application to receive highly pure target cell fractions using MACS. In the future, gene profiling and identification of cell surface markers specific for transplantable cones will be important to define and generate tools for purifying and separating rod or cone photoreceptors for developing stringent cell replacement strategies tackling specific retinal degenerative diseases characterized by rod, cone, or combined cone and rod loss.

## Appendix 1. Gene list

To access the data, click or select the words “Appendix 1.” List of genes that were more than two-fold higher expressed in PN4 photoreceptors as demonstrated by microarray analysis and that were enriched with DAVID for gene ontology term “topological domain: extracellular.” Cdh13 and Kcnv2 were added to the list as they showed up as potentially interesting genes during literature research.
